# Concomitant Bladder Tumor Is a Risk Factor for Bladder Recurrence but Not Upper Tract

**DOI:** 10.3390/curroncol29120727

**Published:** 2022-11-28

**Authors:** Kang Liu, Hongda Zhao, Mario Alvarez-Maestro, Stavros Gravas, Koen Van Renterghem, Guohua Zeng, Chi-Fai Ng, Pilar Laguna, Jeremy Yuen-Chun Teoh, Jean De La Rosette

**Affiliations:** 1 S.H. Ho Urology Centre, Department of Surgery, The Chinese University of Hong Kong, Hong Kong 999077, China; 2 Department of Urology, La Paz University Hospital, 28001 Madrid, Spain; 3 Department of Urology, Faculty of Medicine, School of Health Sciences, University of Thessaly, 41001 Larissa, Greece; 4 Department of Urology, Jessa Hospital University of Hasselt, 3500 Hasselt, Belgium; 5 Department of Urology and Guangdong Key Laboratory of Urology, The First Affiliated Hospital of Guangzhou Medical University, Guangzhou 510000, China; 6 Department of Urology, Medipol Mega University Hospital, Istanbul Medipol University, Istanbul 34000, Turkey

**Keywords:** concomitant bladder tumor, upper tract urothelial carcinoma, kidney-sparing surgery, recurrence-free survival

## Abstract

Objective: To evaluate the clinical outcomes of UTUC patients with or without concurrent bladder tumor. Design, Setting, and Participants: The Clinical Research Office of the Endourology Society-Urothelial Carcinomas of the Upper Tract (CROES-UTUC) Registry included 1134 UTUC patients with or without concurrent bladder tumor treated between 2014 and 2019. Results: In 218 (19.2%) cases, concurrent bladder tumor was present, while in 916 (80.8%) patients, no bladder cancer was found. In the multivariable Cox regression analysis, concomitant bladder tumor (hazard ratio (HR) 1.562, 95% confidence interval (CI) 0.954–2.560, *p* = 0.076) indicated a trend associated with recurrence-free survival for UTUC. Further data dissection confirmed that concomitant bladder tumor is a risk factor of bladder recurrence (HR 1.874, 95% CI 1.104–3.183, *p* = 0.020) but not UTUC recurrence (HR 0.876, 95% CI 0.292–2.625, *p* = 0.812). Kidney-sparing surgery (KSS) (HR 3.940, 95% CI 1.352–11.486, *p* = 0.012), pathological T staging ≥ pT2 (HR 2.840, 95% 1.039–7.763, *p* = 0.042) were significantly associated with UTUC recurrence. KSS does not affect bladder recurrence (HR 0.619, 95% CI 0.242–1.580, *p* = 0.315). A limitation is the retrospective nature of the present study analysis. Conclusions: The presence of concomitant bladder tumor does not increase risk of UTUC recurrence, but it results in an increased risk of bladder recurrence. KSS does not affect bladder recurrence and can still be considered in patients with concomitant bladder tumor.

## 1. Introduction

Urothelial carcinomas are the sixth most common tumors in developed countries [1]. Most of them are in the lower urinary tract while only 5–10% originate from the upper urinary tract [2]. The estimated annual incidence of upper tract urothelial carcinoma (UTUC) ranges from 1 to 2 per 100,000 people [3]. Unlike bladder cancer, which can predominantly be treated by a bladder-sparing endoscopic transurethral resection, UTUC patients are mostly recommended radical nephroureterectomy [4].

UTUC and bladder tumor have many differences not only in tumor pathogenesis and biological behavior, but also in diagnosis, treatment, and prognosis. Unfortunately, in 8–17% of newly onset UTUC cases, concomitant bladder cancer is found [5,6,7,8]. At present, we are still lacking the evidence needed to draw a blueprint of survival outcomes, including overall survival, cancer-specific survival, and recurrence-free survival, in UTUC patients with concurrent bladder tumors.

The Clinical Research Office of the Endourology Society Urothelial Carcinomas of the Upper Tract (CROES-UTUC) registry was established in 2014 [9]. This is one of the largest real-world prospective global datasets on the management of UTUC. Taking this research as an opportunity, we investigated the effects of concomitant bladder tumor on the survival outcomes of UTUC. We believe that this will provide more comprehensive information on the understanding of the role that bladder tumors play in UTUC patients and could help to guide clinical practice.

## 2. Patients and Method

### 2.1. Data Source

The CROES-UTUC registry is a prospective, observational, international, multicenter, cohort study, which enrolled patients with suspected UTUC from 101 centers in 29 countries [9]. The registry follows the recommendations of the Agency for Healthcare Research and Quality for the design and use of patient registries for scientific, clinical, and health policy purposes. The study was registered with clinicaltrials.gov (NCT02281188).

Consecutive patients aged ≥18 years who had suspected UTUC undergoing any type of diagnostic or therapeutic surgical intervention were included. Patients with prior history of bladder cancer were not excluded. The study criteria were wide-ranging to provide comprehensive real-world data regarding the management and outcomes of patients with suspected UTUC.

Clinical data on baseline characteristics, risk factors, clinical assessment, intervention received, and survival outcomes were recorded [10]. Follow-up data were recorded for up to 5 years. Data from all participating centers were collected using an online Data Management System. The Data Management System was a web-based system located and maintained at the CROES Office.

The primary outcomes of this study were overall survival, cancer-specific survival, and recurrence-free survival. Patients were first dichotomized according to whether concurrent bladder tumor was present (concomitant bladder tumor and non-concomitant bladder tumor), and their baseline characteristics were compared.

### 2.2. Statistical Methods

Chi-square tests were used for comparison of categorical variables. Kaplan–Meier analysis was performed for overall survival, cancer-specific survival, and recurrence-free survival; significance was determined by log-rank test. Multivariate Cox regression analyses on overall survival, cancer-specific survival and recurrence-free survival were performed. A *p* < 0.05 was statistically significant. All statistical analyses were performed using SPSS version 25 (IBM Corporation, Armonk, NY, USA).

## 3. Results

### 3.1. Patient Demographics

A total of 1134 patients were included in this analysis, of which 218 patients (19.2%) had concomitant bladder tumor. Table 1 summarizes the clinicopathologic features of the study population: 601 (53.0%) aged more than 70, 814 (71.8%) were male, 738 (65.1%) were smoker or ex-smoker, 704 (62.1%) were ASA I-II, and 258 (22.8%) received 0 on the Charlson Comorbidity Index. Most patients received RNU as the definitive surgical treatment (92.3%). Regarding the tumor pathology, 457 (40.3%) had pTa/Tis/T1 disease, 133 (11.7%) had G1 tumor, and 791 (69.8%) were unifocal tumors. There were 42/218 (19.3%) and 113/916 (12.3%) patients who received adjuvant intravesical instillation.

### 3.2. Comparison between Concomitant and Non-Concomitant Bladder Tumor Patients

While ASA, CCI, pathological T staging and tumor grade showed no differences between the two groups, concomitant bladder tumor group had a high rate of age more than 70 (*p* = 0.047), male gender (*p* = 0.010) and cigarette exposure (*p* = 0.047). By contrast, patients without bladder tumor were more likely to present with unifocal UTUC (*p* = 0.004) and received radical nephroureterectomy (*p* = 0.019).

### 3.3. Recurrence-Free Survival

**Figure 1:**
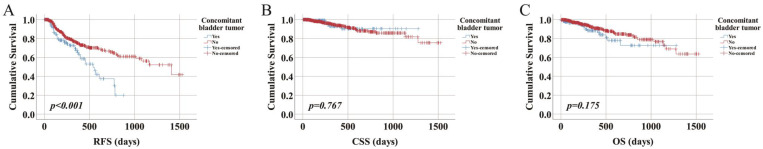
All patients with UTUC and presence or absence of concomitant bladder cancer. (**A**) Recurrence-free survival, (**B**) cancer-specific survival, (**C**) overall survival.

Patients without bladder tumor had better overall recurrence-free survival (RFS) than concomitant bladder cancer patients (*p* < 0.001) (Figure 1A). Although multivariable Cox regression analysis indicated that the only risk factor was multifocal tumor (Hazard ratio (HR) 1.611, 95% confidence interval (CI) 1.017–2.550, *p* = 0.042), concomitant bladder cancer demonstrated a trend of increasing recurrence (HR 1.562, 95% CI 0.954–2.560, *p* = 0.076) (Table 2).

**Figure 2:**
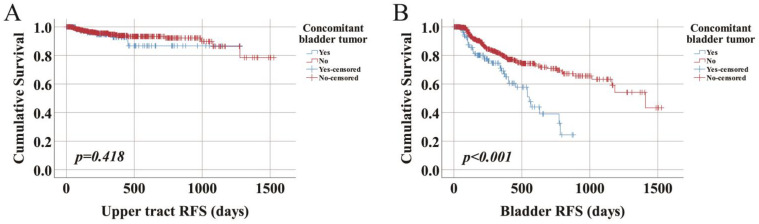
Patients with UTUC and presence or absence of concomitant bladder cancer. (**A**) RFS of the upper urinary tract, (**B**) RFS of the bladder.

Furthermore, we explored what kind of recurrence was affected by concomitant bladder tumors. Of all 158 (13.9%) patients suffering from recurrence, 24 patients presented with upper urinary tract recurrence, 124 patients had bladder cancer recurrence, and 14 patients had recurrence of both. Interestingly, only bladder recurrence, instead of upper urinary tract recurrence, was increased when testing the effect of concomitant bladder tumor status (*p* < 0.001) (Figure 2), which was consistent with the following multivariable Cox regression analysis results. For upper urinary tract recurrence, concomitant bladder tumor was not a risk factor (HR 0.876, 95% CI 0.292–2.625, *p* = 0.812) while the kidney-sparing surgery (KSS) approach (HR 3.940, 95% CI 1.352–11.486, *p* = 0.012) and pathological T staging ≥ pT2 (HR 2.840, 95% CI 1.039–7.763, *p* = 0.042) were independent risk factors (Table 3). However, concomitant bladder tumor was the only independent risk factor (HR 1.874, 95% CI 1.104–3.183, *p* = 0.020) that affected bladder recurrence, irrespective of which surgical approach was taken by patients (KSS: HR 0.619, 95% CI 0.242–1.580, *p* = 0.315) (Table 4).

### 3.4. Cancer-Specific Survival

The Kaplan–Meier plot indicated that concomitant bladder tumor, as a single factor, did not affect cancer-specific survival (*p* = 0.767) (Figure 1B). Multivariable Cox regression analysis confirmed the same result while noting that the tumor T staging ≥ pT2 patients had 5.8 times greater risk of cancer-specific death than those pTa/Tis/T1 patients (Supplementary Table S1).

### 3.5. Overall Survival

We also found that concomitant bladder tumor had no effect on overall survival (*p* = 0.175) (Figure 1B). However, multivariable Cox regression showed that ≥ 70 years was an independent risk factor (HR 2.190, 95% CI 1.033–4.642, *p* = 0.041) (Supplementary Table S2).

## 4. Discussion

Our analyses revealed several remarkable findings. First, since concomitant bladder tumor is an independent risk factor, which increases bladder tumor recurrence in UTUC patients but does not increase upper urinary tract recurrence, urologists should pay more attention to those with concomitant bladder tumor patients during follow-ups, and regularly perform cystoscopy. Second, the upper urinary tract recurrence is associated with higher pathological staging and more conservative surgical approach in UTUC patients, which shows that surgeons should select proper treatment, considering the best interest of the patient. Third, regardless of the co-presence of bladder tumor, cancer-specific survival and overall survival depend more on UTUC tumor stage and patients’ age.

To investigate the impact of concomitant bladder tumor, clinical and molecular differences between bladder cancer and UTUC must be deliberated. In this study, concomitant bladder tumor patients were different from non-concomitant bladder tumor patient’s cohort in terms of their baseline characteristics. Concomitant bladder tumor patients were older, presented more often in male gender and had a higher percentage of cigarette exposure and multifocal tumor but received RNU less often than patients without concomitant bladder tumors. These distribution characteristics might be the result of different biological behaviors and, as such, in line with previous studies [11]. Balkan and East Asian regions suffer from a higher prevalence of UTUC and the reason for UTUC enrichment in these areas has been linked to the high content of arsenic in the drinking water or an exposure to aristolochic acid [3,12,13]. While tobacco smoking and aromatic amines are common risk factors of UTUC and bladder cancer, bladder cancer has unique risk factors, such as pelvic radiotherapy, thiazolidinediones, metabolic disorders, chlorinated drinking water. There are both similarities and differences in pathological and molecular features. For instance, although FGFR3 is common mutation associated with the pathogenesis of both diseases, it is more frequently mutated in UTUC than in bladder cancer patients [14]. With all this evidence, there is no doubt that the biology of patients with both UTUC and bladder cancer is different from patients with solely UTUC.

The impact of simultaneous bladder cancer on patient survival has been explored by several groups. Kuroiwa and colleagues collected data for 2668 nonmetastatic UTUC patients; 23.7% had previous or concomitant bladder cancer. The overall survival was short in patients with previous or concomitant bladder cancer [11]. Zeng et al. focused on cancer-specific survival and found that, after adjustment for covariates, previous or concomitant bladder cancer might have a trend of shortened CSS, although the hazard ratio is small [15]. These findings are different from our findings. The reason that we did not observe a negative correlation between concomitant bladder tumor and cancer-specific survival might be that the proportion of our UTUC patients had a higher pathological T staging (≥ pT2) than other reports. Under these circumstances, UTUC itself becomes the main determining factor of cancer-specific survival. Moreover, while we observed that concomitant bladder tumor is an independent predictor of intravesical recurrence, it is not a predictive factor for overall survival, cancer-specific survival, or upper urinary tract recurrence.

Focusing on the impact on recurrence, Milojevic et al. concluded that prior bladder tumor history had no effect on non-bladder recurrence but was an independent predictor of intravesical recurrence [16]. Pignot and colleagues also conveyed, using a bigger population, that previous or synchronous bladder cancer was an independent prognostic factors for bladder tumor recurrence [17]. Hence, they found that UTUC was more invasive at the time of diagnosis if patients had a concomitant bladder tumor status. To the best of our knowledge, the CROES-UTUC registry enrolled the largest population with concomitant bladder tumor status information, providing a “real-world” perspective to declare concomitant bladder tumor increases as an intravesical recurrence.

Apart from the concomitant situation, in our study, KSS is not a risk factor for intravesical recurrence. Therefore, the bladder is safe in well-selected UTUC patients under KSS. However, while concomitant bladder tumor is not a risk factor for upper urinary tract recurrence, KSS is an independent risk factor for UTUC recurrence, which shows that case selection is extremely crucial. The benefit from KSS for low-risk UTUC includes reduced morbidity associated with radical surgery [18]. Consequently, current EAU guideline strongly recommends offering KSS as primary option only to low-risk UTUC patients [19]. Although concomitant bladder tumor does not affect overall survival, cancer-specific survival, and upper urinary tract recurrence, it requires extra attention during disease management.

In addition, multifocality is a risk factor for overall recurrence in our study, and seems to affect the intravesical recurrence (HR 1.559, 95% CI 0.941–2.581, *p* = 0.085) but not the upper urinary tract (HR 1.741, 95% CI 0.667–4.543, *p* = 0.257). Notably, multifocal status and concomitant bladder tumor are positively correlated. This probably represents some field change effect and underlying biology of the tumor. Chromecki et al. found that tumor multifocality was an independent prognosticator of cancer-specific mortality and disease progression in patients treated with RNU [20]. A high-quality systematic review also revealed that the presence of multifocal tumor is one of the most important disease-related prognostic factors that indicates a worsening outcome in UTUC patients [21]. Accordingly, multifocal tumor patients may need closer follow-up.

The current study has multiple limitations. First, its retrospective design is an inherent limitation. Second, there could be missing data in our cohort that could be compensated for by the significant sample size. Third, it was a heterogenous cohort because of the real-world data collection, which we adjusted for using multivariable analysis. Despite these shortcomings, the strengths are also inherent, as they derive from the global cooperation practice and real-world data, which deliver valuable insights, qhich are needed for better UTUC management.

## 5. Conclusions

The presence of a concomitant bladder tumor does not increase the risk of UTUC recurrence, but results in an increased risk of bladder recurrence. KSS does not affect bladder recurrence, and can still be considered in patients with concomitant bladder tumor.

## Figures and Tables

**Table 1:** Baseline characteristics between groups (concomitant vs. non-concomitant bladder tumor).

Characteristics	Concomitant Bladder Tumor N = 218 (*n*, %)	Non-Concomitant Bladder Tumor N = 916 (*n*, %)	Total N = 1134 (*n*, %)	*p*-Value
Age				
≤70	89 (40.8)	441 (48.1)	530 (46.7)	0.047
>70	129 (59.2)	472 (51.5)	601 (53.0)	
Male gender	172 (78.9)	642 (70.1)	814 (71.8)	0.010
Smoking status				
No	58 (26.6)	301 (32.9)	285 (25.1)	0.047
Yes, Present	50 (22.9)	235 (25.7)	379 (33.4)	
Yes, Past	87 (39.9)	292 (31.9)	359 (31.7)	
ASA				
I	23 (10.6)	119 (13.0)	142 (12.5)	0.115
II	115 (52.8)	447 (48.8)	562 (49.6)	
III	67 (30.7)	319 (34.8)	386 (34.0)	
IV	8 (3.7)	20 (2.2)	28 (2.5)	
V	1 (0.5)	0 (0)	1 (0.1)	
CCI				
0	47 (21.6)	211 (23.0)	258 (22.8)	0.731
1	39 (17.9)	140 (15.3)	179 (15.8)	
2	31 (14.2)	143 (15.6)	174 (15.3)	
3	23 (10.6)	74 (8.1)	97 (8.6)	
4	13 (6.0)	59 (6.4)	72 (6.3)	
5	4 (1.8)	20 (2.2)	24 (2.1)	
6	6 (2.8)	20 (2.2)	26 (2.3)	
7	4 (1.8)	10 (1.1)	14 (1.2)	
8	2 (0.9)	2 (0.2)	4 (0.4)	
9	0 (0)	4 (0.4)	4 (0.4)	
10	0 (0)	1 (0.1)	1 (0.1)	
Procedure				
RNU performed	193 (88.5)	854 (93.2)	1047 (92.3)	0.019
KSS performed	25 (11.5)	62 (6.8)	87 (7.7)	
Tumor stage (2009)				
pTa	44 (20.2)	165 (18.0)	209 (18.4)	0.789
pTis	5 (2.3)	13 (1.4)	18 (1.6)	
pT1	39 (17.9)	191 (20.9)	230 (20.3)	
pT2	37 (17.0)	166 (18.1)	203 (17.9)	
pT3	56 (25.7)	259 (28.3)	315 (27.8)	
pT4	6 (2.8)	31 (3.4)	37 (3.3)	
Tumor grade				
G1	26 (11.9)	107 (11.7)	133 (11.7)	0.342
G2	42 (19.3)	230 (25.1)	272 (24.0)	
G3	108 (49.5)	465 (50.8)	573 (50.5)	
GX	4 (1.8)	8 (0.9)	12 (1.1)	
Multifocal tumor				
No	141 (64.7)	650 (71.0)	791 (69.8)	0.004
Yes	60 (27.5)	166 (18.1)	226 (19.9)	

ASA: American Society of Anesthesiologists; CCI: Charlson Comorbidity Index; RNU: Radical nephroureterectomy; KSS: Kidney-sparing surgery.

**Table 2:** Multivariate Cox regression analyses of recurrence-free survival.

Characteristics	HR (95% CI)	*p*-Value
Concomitant bladder tumor		
No	Reference	
Yes	1.562 (0.954–2.560)	0.076
Age		
<70	Reference	
≥70	1.118 (0.728–1.718)	0.611
Gender		
Female	Reference	
Male	0.665 (0.383–1.154)	0.147
Smoking status		
No	Reference	
Yes	1.318 (0.778–2.234)	0.305
ASA		
I-II	Reference	
III-V	1.088 (0.706–1.676)	0.703
CCI		
0	Reference	
1–2	0.771 (0.465–1.278)	0.313
3–10	1.302 (0.753–2.251)	0.346
Procedure		
RNU	Reference	
KSS	1.120 (0.547–2.293)	0.756
Tumor stage		
<pT2	Reference	
≥pT2	1.276 (0.793–2.055)	0.315
Tumor grade		
G1	Reference	
G2	0.942 (0.490–1.812)	0.859
G3	0.600 (0.305–1.180)	0.139
Multifocal tumor		
No	Reference	
Yes	1.611 (1.017–2.550)	0.042

ASA: American Society of Anesthesiologists; CCI: Charlson Comorbidity Index; RNU: Radical nephroureterectomy; KSS: Kidney-sparing surgery.

**Table 3:** Multivariate Cox regression analyses on recurrence-free survival of upper urinary tract.

Characteristics	HR (95% CI)	*p*-Value
Concomitant bladder tumor		
No	Reference	
Yes	0.876 (0.292–2.625)	0.812
Age		
<70	Reference	
≥70	0.811 (0.346–1.900)	0.629
Gender		
Female	Reference	
Male	0.930 (0.301–2.871)	0.900
Smoking status		
No	Reference	
Yes	1.473 (0.515–4.214)	0.470
ASA		
I-II	Reference	
III-V	0.749 (0.316–1.779)	0.513
CCI		
0	Reference	
1–2	0.352 (0.117–1.062)	0.064
3–10	1.285 (0.487–3.390)	0.613
Procedure		
RNU	Reference	
KSS	3.940 (1.352–11.486)	0.012
Tumor stage		
<pT2	Reference	
≥pT2	2.840 (1.039–7.763)	0.042
Tumor grade		
G1	Reference	
G2	1.542 (0.308–7.718)	0.598
G3	0.885 (0.165–4.737)	0.886
Multifocal tumor		
No	Reference	
Yes	1.741 (0.667–4.543)	0.257

ASA: American Society of Anesthesiologists; CCI: Charlson Comorbidity Index; RNU: Radical nephroureterectomy; KSS: Kidney-sparing surgery.

**Table 4:** Multivariate Cox regression analyses on recurrence-free survival of bladder.

Characteristics	HR (95% CI)	*p*-Value
Concomitant bladder tumor		
No	Reference	
Yes	1.874 (1.104–3.183)	0.020
Age		
<70	Reference	
≥70	1.288 (0.796–2.084)	0.303
Gender		
Female	Reference	
Male	0.646 (0.353–1.180)	0.155
Smoking status		
No	Reference	
Yes	1.225 (0.690–2.175)	0.488
ASA		
I-II	Reference	
III-V	1.040 (0.646–1.673)	0.872
CCI		
0	Reference	
1–2	0.816 (0.465–1.430)	0.477
3–10	1.357 (0.742–2.483)	0.322
Procedure		
RNU	Reference	
KSS	0.619 (0.242–1.580)	0.315
Tumor stage		
<pT2	Reference	
≥pT2	1.043 (0.622–1.749)	0.874
Tumor grade		
G1	Reference	
G2	0.861 (0.424–1.749)	0.679
G3	0.615 (0.296–1.278)	0.193
Multifocal tumor		
No	Reference	
Yes	1.559 (0.941–2.581)	0.085

ASA: American Society of Anesthesiologists; CCI: Charlson Comorbidity Index; RNU: Radical nephroureterectomy; KSS: Kidney-sparing surgery.

## Data Availability

Not applicable.

## Supplementary Materials

The following supporting information can be downloaded at: https://www.mdpi.com/article/10.3390/curroncol29120727/s1, Table S1: Multivariate Cox regression analyses on cancer-specific survival; Table S2: Multivariate Cox regression analyses on overall survival.
