# Supplementary material for: Concomitant Bladder Tumor Is a Risk Factor for Bladder Recurrence but Not Upper Tract
Source: Curr Oncol. 2022 Nov 28;29(12):9284–93. doi: 10.3390/curroncol29120727 (PMC9776685; doi:10.3390/curroncol29120727)
Supplement: Supplementary file 1 [file curroncol-29-00727-s001.zip › curroncol-2006479-supplementary.pdf]

Table S1. Multivariate Cox regression analyses on cancer-specific survival.

**Characteristics**	**HR (95%CI)**	***p*-value**
Concomitant bladder tumor		
No	Reference	
Yes	1.073 (0.293-3.928)	0.915
Age		
<70	Reference	
≥70	2.373 (0.867-6.499)	0.093
Gender		
Female	Reference	
Male	0.785 (0.258-2.393)	0.670
Smoking status		
No	Reference	
Yes	1.595 (0.530-4.797)	0.406
ASA		
I-II	Reference	
III-V	1.824 (0.747-4.453)	0.187
CCI		
0	Reference	
1-2	1.122 (0.375-3.354)	0.837
3-10	1.450 (0.443-4.744)	0.539
RNU		
No	Reference	
Yes	<0.001 (<0.001-1.429E+227)	0.964
Tumor stage		
<pT2	Reference	
≥pT2	5.851 (1.315-26.041)	0.020
Tumor grade		
G1	Reference	
G2	<0.001 (<0.001-7.794E+141)	0.948
G3	2.626 (0.326-21.178)	0.365
Multifocal tumor		
No	Reference	
Yes	0.638 (0.242-1.681)	0.363

ASA: American Society of Anesthesiologists; CCI: Charlson Comorbidity Index; RNU: Radical nephroureterectomy.

Table S2. Multivariate Cox regression analyses on overall survival.

Characteristics	HR (95%CI)	*p*-value
Concomitant bladder tumor		
No	Reference	
Yes	2.008 (0.944-4.269)	0.070
Age		
<70	Reference	
≥70	2.190 (1.033-4.642)	0.041
Gender		
Female	Reference	
Male	1.048 (0.426-2.581)	0.918
Smoking status		
No	Reference	
Yes	0.947 (0.426-2.106)	0.895
ASA		
I-II	Reference	
III-V	1.695 (0.859-3.345)	0.128
CCI		
0	Reference	
1-2	1.606 (0.653-3.954)	0.302
3-10	2.239 (0.853-5.874)	0.102
RNU		
No	Reference	
Yes	0.214 (0.028-1.642)	0.138
Tumor stage		
<pT2	Reference	
≥pT2	1.902 (0.902-4.010)	0.091
Tumor grade		
G1	Reference	
G2	1.001 (0.198-5.067)	0.999
G3	2.465 (0.555-10.945)	0.236
Multifocal tumor		
No	Reference	
Yes	0.870 (0.430-1.758)	0.698

ASA: American Society of Anesthesiologists; CCI: Charlson Comorbidity Index; RNU: Radical nephroureterectomy.
